# Identification of β-Lapachone as a Potent USP22 Inhibitor That Suppresses Cancer Stemness and Enhances Chemosensitivity in Lung Adenocarcinoma

**DOI:** 10.3390/ijms27125561

**Published:** 2026-06-19

**Authors:** Yuanyuan Gao, Keqiang Zhang, Wendong Li, John Liu, David Kwon, Lilian Gu, Aimin Li, Hongwei Holly Yin, Claudia Kowolik, Mahima Raul, David A. Horne, Dan J. Raz

**Affiliations:** 1 Division of Thoracic Surgery, City of Hope National Medical Center, Duarte, CA 91010, USA; 2 School of Basic Medicine, Ningxia Medical University, Yinchuan 750004, China; 3 Cancer Biology and Molecular Medicine, City of Hope Beckman Research Institute, Duarte, CA 91010, USA; 4 Pathology Core of Shared Resources, City of Hope National Medical Center, Duarte, CA 91010, USA

**Keywords:** ubiquitin-specific protease 22 (USP22), β-Lapachone, deubiquitinase inhibitor, cancer stem cells, chemosensitivity, lung adenocarcinoma (LUAD)

## Abstract

Ubiquitin-specific protease 22 (USP22) regulates epigenetic gene expression by deubiquitinating histone H2B (H2Bub1) and upregulating oncogenic proteins and pathways, while antagonizing p53-mediated tumor suppression. USP22 is frequently overexpressed in cancers and associated with therapy resistance and poor prognosis yet remains largely untargeted pharmacologically. Here, using a fluorescence-based USP22 deubiquitinase assay to screen the LOPAC^®1280^ library, we identified β-Lapachone, a natural ortho-naphthoquinone with strong anticancer activities, as a potent USP22 inhibitor. β-Lapachone potently inhibited USP22 enzymatic activity, with a half-maximal inhibitory concentration (IC_50_) of ~0.75 μM, and molecular docking revealed its occupation of the catalytic pocket adjacent to the USP22 active-site triad, supporting a potential binding mode. Functionally, β-Lapachone suppressed proliferation and induced apoptosis in A549 and H1299 RAS-mutant lung adenocarcinoma (LUAD) cells, while USP22 knockout conferred marked resistance, indicating partial USP22 dependence. In patient-derived LUAD models, β-Lapachone inhibited sphere formation and reduced CD133^+^ cancer stem cell populations. Notably, it synergized with cisplatin to enhance DNA damage and apoptosis. In vivo, β-Lapachone significantly suppressed tumor growth in a syngeneic KRAS-mutant/p53-Null mouse lung cancer model and further potentiated cisplatin-induced antitumor effects. Collectively, these findings identify β-Lapachone as a potent inhibitor of USP22 and validate USP22 inhibition as a key mechanism underlying its anticancer activity in LUAD cells, both in vitro and in vivo.

## 1. Introduction

The ubiquitin–proteasome system (UPS) is a central regulator of protein homeostasis, controlling the stability, localization, and function of more than 80% of cellular proteins. Through this activity, the UPS governs fundamental biological processes including cell growth, survival, transcription, DNA damage repair, and immune regulation. Dysregulation of the UPS has been implicated in the pathogenesis of numerous human diseases, particularly cancer, where aberrant ubiquitination drives oncogenic signaling, therapeutic resistance, and immune evasion [1,2]. Protein ubiquitination is a highly dynamic and reversible post-translational modification, mediated by the coordinated actions of E1, E2, and E3 enzymes and reversed by deubiquitinating enzymes (DUBs) [3,4]. The DUB family comprises more than 100 members classified into six subfamilies, among which ubiquitin-specific proteases (USPs) constitute the largest group [3]. Increasing evidence indicates that dysregulated USPs contribute to carcinogenesis by regulating cell proliferation, survival, and transcriptional programs, making them attractive targets for anticancer therapy despite partial functional redundancy within the DUB family [4,5].

Ubiquitin-specific protease 22 (USP22) has emerged as a particularly important oncogenic DUB with diverse roles in cancer progression and immune regulation. USP22 is the catalytic subunit of the deubiquitination module (DubM) of the Spt-Ada-Gcn5 acetyltransferase (SAGA) complex, where it removes monoubiquitin from histone H2B (H2Bub1) and histone H2A, thereby promoting transcriptional activation of genes essential for cell proliferation and survival [6,7,8]. USP22 was originally identified as part of the “death-from-cancer” 11-gene signature [9], and its overexpression correlates with poor prognosis in multiple tumor types, including breast, colon, and lung cancers [6,10,11]. Beyond its epigenetic function, USP22 regulates key oncogenic pathways by stabilizing or modulating proteins such as c-Myc, Sirtuin 1 (SIRT1), cyclin D1, mechanistic target of rapamycin (mTOR) complex 1 (mTORC1), and p53, thereby broadly enhancing tumorigenic signaling [6,12,13,14,15]. USP22 stabilizes Yes-associated protein (YAP), promotes melanoma cell growth, and promotes resistance to the BRAF inhibitor [16]. USP22 is also a critical mediator of the DNA damage response, required for gamma-H2A.X (γH2A.X) deposition and efficient double-strand break repair through interaction with partner and localizer of BRCA2 (PALB2) [17,18]. Consistent with these functions, USP22 overexpression promotes chemoresistance, cancer stemness, angiogenesis, and metabolic reprogramming across multiple cancers [9,19,20,21,22,23].

Recent studies further demonstrate USP22 as an important regulator of tumor immune evasion. In KRAS-mutant lung adenocarcinoma (LUAD), oncogenic KRAS signaling induces immunosuppression in part by elevating programmed death-ligand 1 (PD-L1) expression [24]. USP22 directly deubiquitinates and stabilizes PD-L1 on tumor cells, thereby strengthening cancer cell communication with the tumor microenvironment and promoting immune suppression and tumor persistence [25,26,27]. Our previous work demonstrated that genetic depletion of USP22 in KRAS-mutant LUAD suppresses cancer stem cell maintenance, inhibits angiogenesis, and reduces tumor growth and metastasis, particularly in KRAS and liver kinase B1 (LKB1) co-mutant models [18,28,29]. Importantly, loss of USP22 markedly sensitizes KRAS-mutant LUAD to cisplatin chemotherapy and irradiation, independent of p53 status [18,28,29]. Together, these findings establish USP22 as a compelling therapeutic target capable of simultaneously suppressing tumor-intrinsic oncogenic signaling and tumor-extrinsic immune evasion.

Despite its strong therapeutic potential, pharmacological inhibition of USP22 remains challenging. Similarly to the orthologues of yeast complex, human USP22 is a component of the SAGA DubM that contains ATXN7, ATXN7L3, and ENY2, functioning as part of the intact complex and lacks catalytic activity when isolated, complicating the development of conventional small-molecule inhibitors [30,31,32,33].To date, only a limited number of experimental USP22 inhibitors have been reported, including macrocyclic peptides targeting the USP22 DubM [34] and early small-molecule compounds with immunomodulatory activity [35]. Recent studies identified two selective and potent USP22 inhibitors Rottlerin [36], Morusin [36] and Gentiopicroside [37]. Another study, using the virtual screening of FDA-approved compounds from DrugBank, revealed that Ergotamine showed high binding affinities to USP22 and suppressed USP22 activity [38]. Thus, identification of potent, specific, and mechanistically validated USP22 inhibitors remains an unmet need.

In this study, we identify β-Lapachone, a naturally occurring ortho-naphthoquinone originally isolated from *Handroanthus impetiginosus*, as a potent and previously unrecognized inhibitor of USP22. β-Lapachone has demonstrated broad anticancer activity against multiple tumor types, including lung, pancreatic, breast, colon, and melanoma [39,40]. Its antitumor effects have been attributed primarily to its function as a topoisomerase I inhibitor, which was one of its first mechanisms reported against prostate and breast cancer [41,42,43]. NAD(P)H-quinone oxidoreductase 1 (NQO1) dependent redox cycling [44], induction of DNA damage, inhibition of DNA repair, and modulation of oncogenic pathways such as mTOR and AKT signaling [45,46]. β-Lapachone exhibits potent cytotoxicity at micromolar concentrations, synergizes with chemotherapy and radiotherapy, and has advanced into phase II clinical trials for pancreatic cancer with a favorable toxicity profile [47]. However, the full spectrum of its molecular targets remains incompletely understood.

This finding of β-Lapachone as a potent inhibitor of USP22 expands the known target landscape of β-Lapachone and provides a new mechanistic basis for its anticancer and immunomodulatory effects. Using KRAS-mutant lung cancer models in which USP22 is highly overexpressed, including in vitro 2D cultures and in vivo subcutaneous KRAS-mutant lung cancer, we demonstrate that β-Lapachone, as a potent USP22 inhibitor, efficiently suppresses tumor growth and synergistically enhances cisplatin efficacy. Importantly, these effects are mediated, at least in part, through inhibition of USP22. Together, our results establish USP22 as a novel functional target of β-Lapachone and support its therapeutic potential for KRAS-mutant LUAD, by simultaneously targeting tumor growth, therapy resistance, and immune evasion.

## 2. Results

### 2.1. Identification of β-Lapachone as a Potent USP22 Inhibitor

**Figure 1:**
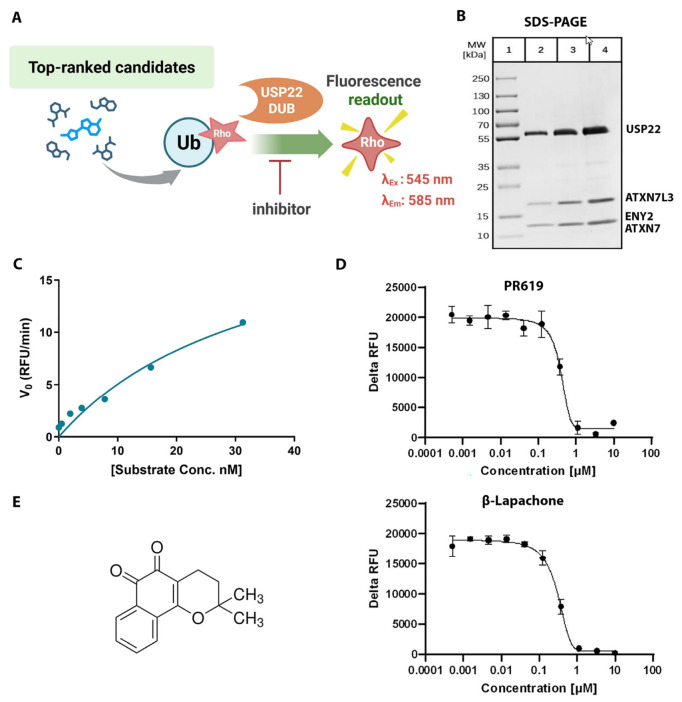
Identification of β-Lapachone as a USP22 inhibitor by biochemical DUB screening. (**A**) Schematic of the USP22 enzymatic assay using ubiquitin–rhodamine (Ub-Rho) substrate cleavage and fluorescence detection (λEx 545 nm/λEm 585 nm). (**B**) SDS-PAGE showing purified recombinant USP22 DubM (lane 1: protein marker, lane 2–4: 1, 2, and 3 μg of purified DubM protein, respectively) with subunits USP22 (60.2 kDa), ENY2 (11.5 kDa), ATXN7L3 (17.1 kDa), and ATXN7 (11.4 kDa). (**C**) Michaelis–Menten kinetic analysis of enzyme activity showing a Vmax of 22.95 and a Km of 35.51 nM. (**D**) Dose–response inhibition curve of the positive control PR619 validating assay performance. (**E**) Chemical structure of β-Lapachone and its dose-dependent inhibition of USP22 (IC_50_ ≈ 0.75 μM).

A fluorescence-based enzymatic assay was established Ub-Rho as the substrate, in which cleavage of Ub-Rho by USP22 generates a measurable fluorescence signal (λEx 545 nm/λEm 585 nm) (Figure 1A). Recombinant human USP22 DubM, composed of USP22, ENY2, ATXN7L3, and ATXN7, was expressed and reconstituted for biochemical characterization and inhibitor screening. The purity of the recombinant USP22 complex was verified by SDS-PAGE and confirmed by Western blot analysis, which showed distinct bands corresponding to USP22 (60.2 kDa), ATXN7L3 (17.1 kDa), ENY2 (11.5 kDa), and ATXN7 (11.4 kDa) (Figure 1B). Michaelis–Menten analysis revealed a Vmax of 22.95 and a Km of 35.51 nM, indicating that the enzyme exhibits high apparent affinity for the substrate, reaching half-maximal velocity at ~35 nM substrate concentration (Figure 1C). To validate assay performance and establish a reference for inhibition, PR619, a well-characterized pan-deubiquitinase (pan-DUB) inhibitor, was used as a positive control and produced a robust dose-dependent inhibition curve with a half-maximal inhibitory concentration (IC_50_) of 0.41 μM, confirming the sensitivity and dynamic range of the assay (Figure 1D). Using this validated platform, the reconstituted USP22 DubM was screened against the Library of Pharmacologically Active Compounds (LOPAC^®1280^, Sigma-Aldrich, St. Louis, MO, USA) to identify potential inhibitors. Among the top candidates, β-Lapachone emerged as a potent inhibitor of USP22. Dose–response measurements showed that β-Lapachone inhibited USP22 deubiquitinase activity in a concentration-dependent manner with an IC_50_ of approximately 0.75 μM, demonstrating inhibitory potency comparable to the positive control PR619 (Figure 1E).

### 2.2. β-Lapachone Suppresses Lung Cancer Cell Proliferation in a USP22-Dependent Manner

**Figure 2:**
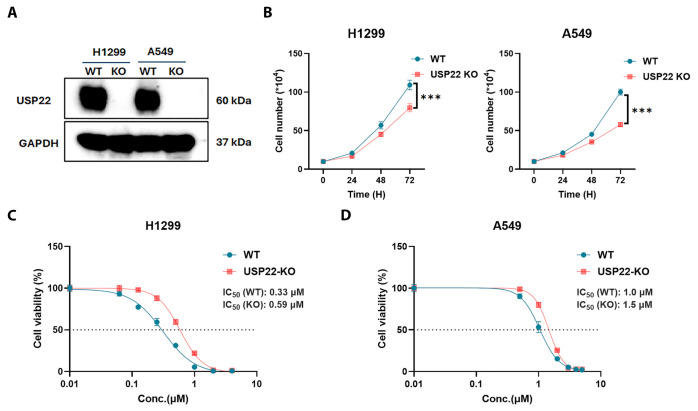
β-Lapachone suppresses cell proliferation in a partially USP22-dependent manner in lung cancer cells. (**A**) Immunoblot confirming USP22 depletion in H1299 and A549 USP22-knockout (KO) cells compared with wild-type (WT) controls. GAPDH served as a loading control. (**B**) Proliferation curves of WT and USP22-KO A549 and H1299 cells, *** *p* < 0.001, USP22 KO vs. WT. Dose–response curves of β-Lapachone in WT and (**C**) USP22-KO H1299 and (**D**) USP22-KO A549 cells. The dashed line indicates 50% viability.

We established USP22-knockout (KO) cell lines in H1299 and A549 lung cancer cells. Immunoblot analysis confirmed depletion of USP22 protein in KO cells relative to wild-type (WT) controls in both cell lines, validating the genetic model (Figure 2A). Under basal conditions, USP22 deletion is not lethal to cancer cells, while USP22-KO cells exhibited significantly reduced proliferation compared to WT counterpart cells over a 72-h time course in both A549 and H1299 lines, suggesting that USP22 is required for optimal lung cancer cell proliferation (Figure 2B). To evaluate whether USP22 contributes to the antiproliferative effects of β-Lapachone and to further assess its activity as a potential USP22 inhibitor in cancer cells, we compared the responses of WT and USP22-KO cells following treatment. Cell viability was measured across a range of β-Lapachone concentrations in WT and KO cells. β-Lapachone reduced cell viability in a concentration-dependent manner in WT H1299 and A549 cells (Figure 2C,D). Notably, USP22-KO cells exhibited significantly reduced sensitivity to β-Lapachone compared to their WT counterparts, as reflected by a rightward shift in the dose–response curve and increased IC_50_ values (H1299: WT 0.33 μM vs. KO 0.59 μM; A549: WT 1.0 μM vs. KO 1.5 μM). These results support USP22 as a functionally relevant target of β-Lapachone in cancer cells, although additional targets may also contribute to its antiproliferative activity. Taken together, these results demonstrate that the antiproliferative activity of β-Lapachone is at least partially dependent on USP22 expression, supporting USP22 as a direct functional target in lung cancer cells.

### 2.3. β-Lapachone Inhibits USP22-Driven Oncogenic Signaling and Induces Apoptosis, DNA Damage in Lung Cancer Cells

**Figure 3:**
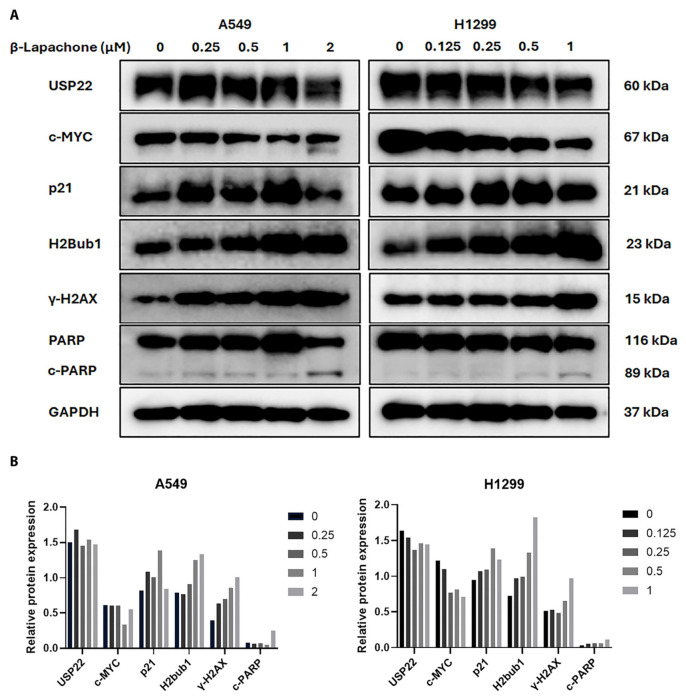
β-Lapachone modulates USP22-associated signaling pathways. (**A**) Representative Western blot analyses and (**B**) densitometric quantification of USP22 and its downstream signaling targets in A549 and H1299 cells treated with increasing concentrations of β-Lapachone for 48 h. Protein levels of c-Myc were decreased, whereas p21, H2Bub1, γ-H2A.X, and cleaved PARP (c-PARP) were increased. GAPDH served as a loading control.

To further evaluate the inhibition of USP22 by β-Lapachone in cancer cells and to investigate the molecular mechanisms underlying its anticancer effects, we analyzed USP22-associated downstream signaling in A549 and H1299 lung cancer cells. Western blot analysis showed that β-Lapachone treatment did not substantially reduce USP22 protein levels (Figure 3A). However, it increased H2Bub1, the authentic substrate of USP22, and markedly suppressed USP22-driven oncogenic signaling, as evidenced by decreased c-Myc protein (an important target of USP22) in both LUAD cells (Figure 3A). These findings suggest that β-Lapachone acts as a functional inhibitor of USP22 activity rather than a regulator of its protein stability. USP22 has been reported to antagonize p53 transcriptional activation by deubiquitinating Sirtuin 1 (SIRT1) deacetylase, which catalyzes the deacetylation of p53 [12]. Consistent with reactivation of the p53 pathway following USP22 inhibition, β-Lapachone treatment significantly upregulated p21, a cyclin-dependent kinase inhibitor and well-established transcriptional target of p53 in p53-wild-type A549 lung cancer cells. Interestingly, β-Lapachone also upregulates p21 expression in p53-deficient H1299 cells, suggesting that p21 upregulation can occur through both p53-dependent and p53-independent mechanisms, and induces cell cycle arrest and apoptosis regardless of p53 status in cancer cells, as reported previously [48,49]. In addition, consistent with our previous finding that USP22-KO impairs DNA damage repair, the accumulation of γ-H2A.X, a marker of DNA double-strand breaks, together with increased PARP cleavage, indicated that β-Lapachone induces DNA damage and apoptosis in lung cancer cells (Figure 3A), indicating these effects should be partially associated with the inhibition of USP22 by β-Lapachone. Densitometric quantification of the Western blot analysis further demonstrated that β-Lapachone treatment drastically modulated the expression of these proteins in these two cancer cells (Figure 3B). These data show that β-Lapachone modulates USP22-regulated signaling, supporting USP22 as a key functional target of its anticancer activity. Collectively, our findings indicate that β-Lapachone exerts its effects, at least in part, by inhibiting USP22 signaling, leading to suppression of c-Myc-driven transcription, DNA damage and apoptosis in cancer cells.

### 2.4. β-Lapachone Suppresses Lung Cancer Stem-like Properties and Self-Renewal Through Inhibition of USP22 Signaling

**Figure 4:**
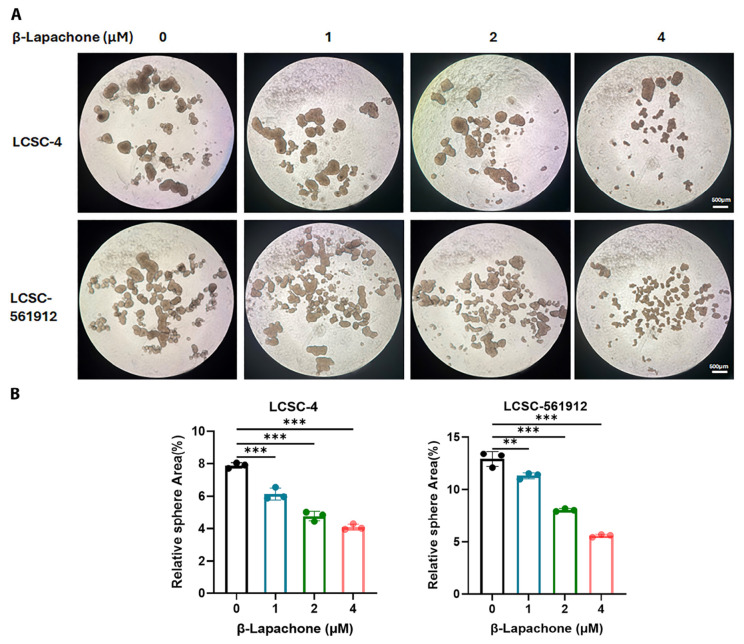

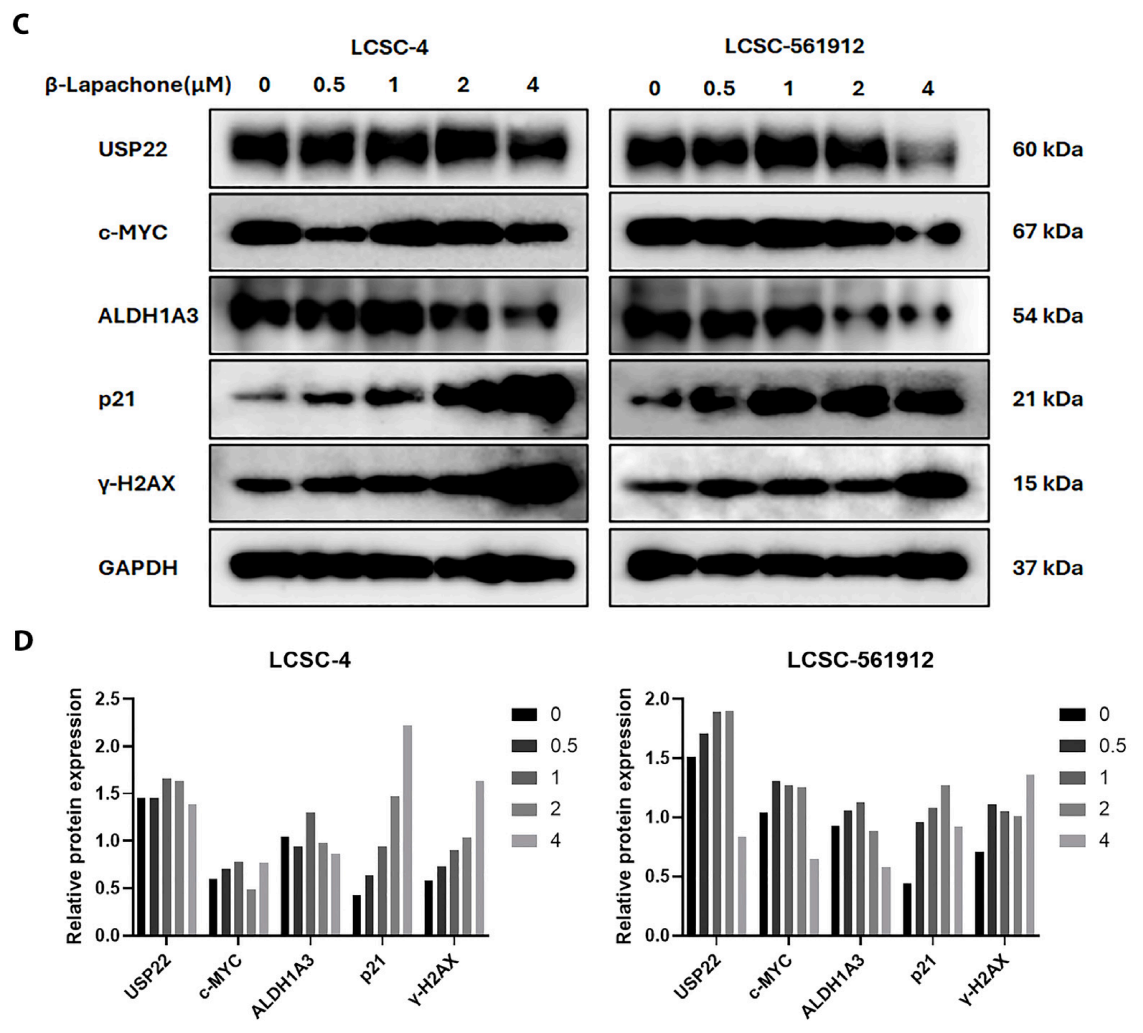
Effects of β-Lapachone on lung cancer stemness and self-renewal. (**A**) Representative images (4× objective; scale bar, 500 μM) of sphere formation assays in patient-derived lung cancer stem-like cell lines LCSC-4 and LCSC-561912 treated with increasing concentrations of β-Lapachone (0–4 μM) under serum-free, non-adherent conditions. (**B**) Sphere area was quantified using ImageJ Version 1.54s and normalized to untreated controls, ** *p* < 0.01, *** *p* < 0.001, β-Lapachone vs. untreated control. (**C**) Representative Western blot analyses and (**D**) Densitometric quantification of indicated proteins in LCSC-4 and LCSC-561912 cells treated with β-Lapachone.

We previously reported that USP22 is enriched in human CD133^+^ cancer-initiating cells and promotes cancer stem cell maintenance and cisplatin resistance in LUAD, partly through regulation of ALDH1A3 [29]. Herein, to determine whether β-Lapachone impairs cancer stem-like properties partially through USP22 inhibition, we performed sphere formation assays in patient-derived lung cancer stem-like cell lines LCSC-4 and LCSC-561912 [29]. β-Lapachone reduced sphere formation in a concentration-dependent manner in both models, as quantified by decreased sphere area (Figure 4A,B). Because control spheres frequently fused into larger aggregates during culture, sphere area was used as the primary quantitative readout in lieu of sphere number, providing a more reliable measure of self-renewal capacity. These findings indicate that β-Lapachone impairs the self-renewal capacity of lung cancer stem-like cells. At the molecular level, β-Lapachone reduced the expression of stemness-associated markers c-Myc and ALDH1A3, which are also important targets of USP22, while concurrently upregulating p21 and γ-H2A.X (Figure 4C). Densitometric quantification of the Western blot analysis is shown in Figure 4D. USP22 protein levels exhibited modest or cell line-dependent changes, suggesting that β-Lapachone primarily suppresses stem-like phenotypes through functional inhibition of USP22 activity rather than uniform downregulation of its protein abundance. Collectively, these data demonstrate that β-Lapachone effectively targets lung cancer stemness and self-renewal capacity, likely through disruption of USP22-driven oncogenic signaling, supporting its potential to suppress tumor-initiating cell populations.

### 2.5. β-Lapachone Enhances Cisplatin-Induced DNA Damage, Apoptosis, and Clonogenic Suppression in Lung Cancer Cells

**Figure 5:**
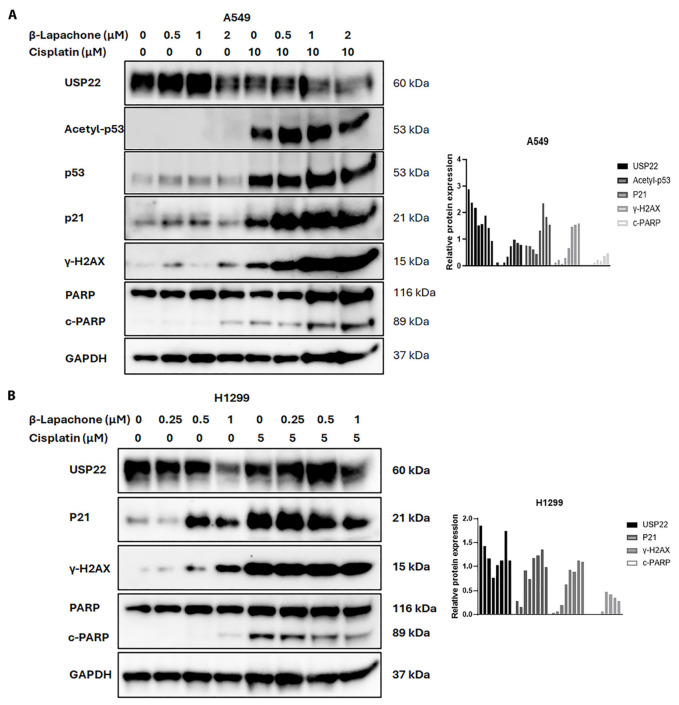

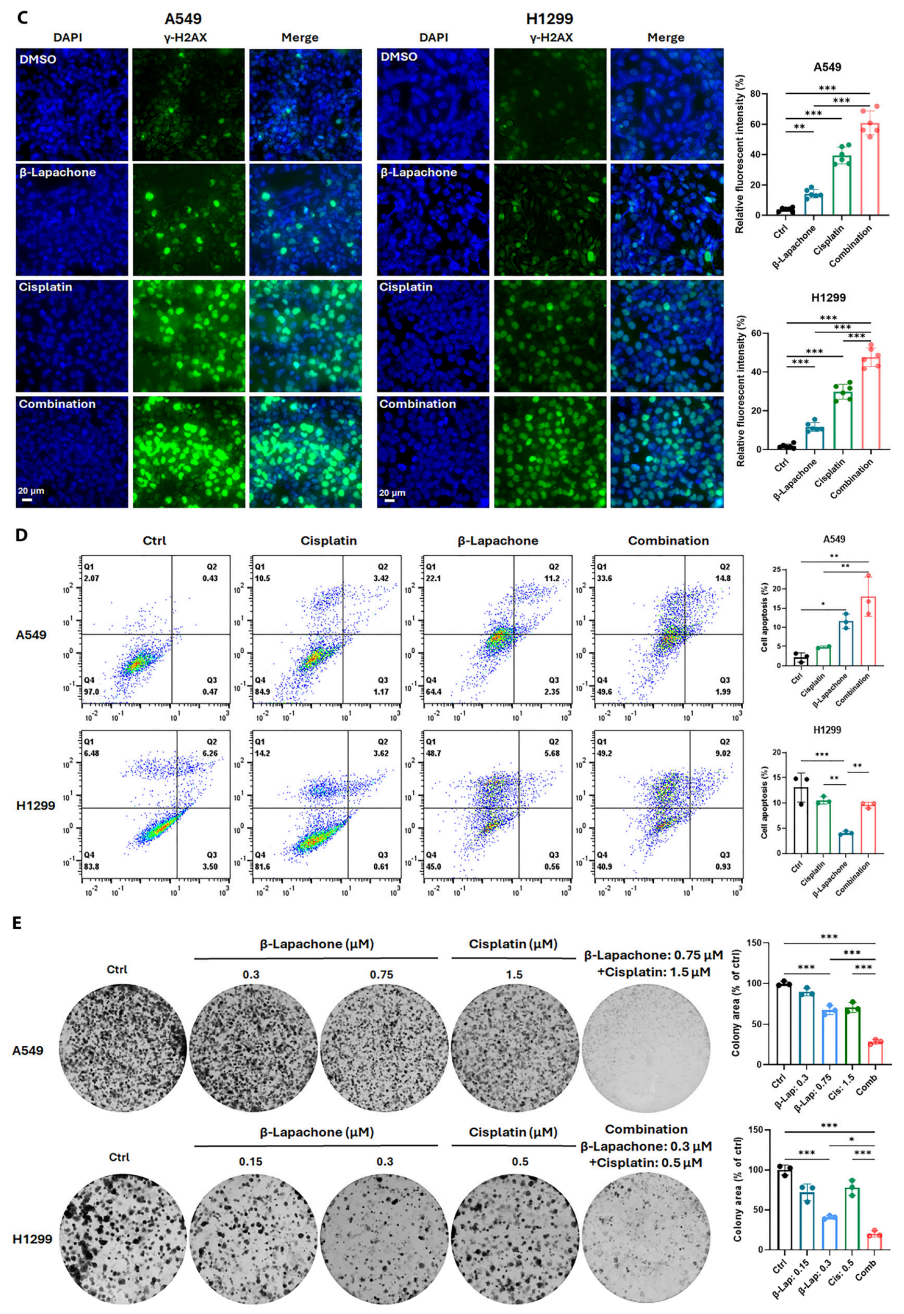
β-Lapachone enhances cisplatin-induced DNA damage, apoptosis, and clonogenic suppression in lung cancer cells. Representative Western blot analyses (left panel) and densitometric quantification (right panel) of indicated proteins in (**A**) A549 and (**B**) H1299 cells treated with β-Lapachone, cisplatin, or their combination for 48 h. γ-H2A.X and cleaved PARP, total p53 and acetylated p53 (Lys382) in A549 were increased in the combination group compared with single-agent treatments. (**C**) Immunofluorescence staining of γ-H2A.X (green) and DAPI (blue) showing representative γ-H2A.X, DAPI, and merged images (left panel). Quantification of γ-H2A.X foci (right panel) demonstrated that β-Lapachone induced DNA damage and further enhanced cisplatin-induced DNA damage in A549 and H1299 cells, scale bar: 20 μM. (**D**) Flow cytometric analysis of apoptosis in cisplatin and β-Lapachone treated A549 and H1299 cells, demonstrating enhanced combine efficiency and increased apoptotic rates, * *p* < 0.05, ** *p* < 0.01, *** *p* < 0.001, β-Lapachone/Cisplatin vs. untreated control or combination. (**E**) Clonogenic assays in A549 and H1299 cells treated with β-Lapachone, cisplatin, or their combination for 10–14 days. Representative crystal violet-stained colonies are shown. Colony area was quantified using ImageJ and normalized to untreated controls, * *p* < 0.05, *** *p* < 0.001, β-Lapachone treated vs. untreated control.

We previously demonstrated that knockdown of USP22 in cancer cells significantly enhances sensitivity to both radiotherapy and cisplatin chemotherapy, likely through its involvement in DNA damage repair [18,28,29]. To evaluate whether β-Lapachone potentiates the anticancer efficacy of cisplatin, we examined the combined effects of the two agents on DNA damage, apoptosis, cell cycle regulation, and clonogenic survival in lung cancer cells. Western blot analysis demonstrated that combination treatment markedly increased γ-H2A.X accumulation and PARP cleavage in both A549 cells (Figure 5A) and H1299 cells (Figure 5B) compared with either single agent alone, indicating enhanced DNA double-strand break induction and apoptotic activation (Figure 5A,B). p21 expression was further elevated in the combination group relative to monotherapy, suggesting augmented cell cycle arrest by the combination. Consistent with the enhanced apoptotic response, β-Lapachone alone slightly upregulated total and acetylated p53 protein levels, while significantly enhancing cisplatin-induced p53 expression and acetylation in A549 lung cancer cell (Figure 5A), suggesting an enhancement of p53 tumor-suppressor activity by the combination treatment. To further validate the synergistic effect of the combination, DNA damage and apoptosis were assessed by γ-H2A.X immunofluorescence and flow cytometry, respectively. As shown in Figure 5C, β-Lapachone significantly enhanced cisplatin-induced γ-H2A.X foci formation in both A549 and H1299 cells, indicating increased DNA double-strand break accumulation. Consistent with these findings, flow cytometric analysis revealed that the combination of β-Lapachone (1 μM) with cisplatin (5 μM) treatment induced substantially higher apoptotic rates than either agent alone in both cell lines (Figure 5D). These results collectively indicate that β-Lapachone potentiates the anticancer activity of cisplatin by enhancing DNA damage and promoting apoptosis.

Clonogenic survival assays corroborated these findings, demonstrating that combination treatment produced a significantly greater reduction in colony-forming capacity compared with either β-Lapachone or cisplatin alone (Figure 5E). Quantification of relative colony area confirmed that β-Lapachone potentiates the long-term antiproliferative effect of cisplatin. Collectively, these results demonstrate that β-Lapachone synergistically enhances cisplatin-induced DNA damage and apoptosis, leading to greater suppression of clonogenic growth in lung cancer cells. Notably, these effects resemble the enhanced sensitivity to cisplatin observed in USP22-knockout cancer cells, further supporting the functional relevance of USP22 inhibition in mediating the response.

### 2.6. β-Lapachone Potentiates Cisplatin Antitumor Efficacy In Vivo

**Figure 6:**
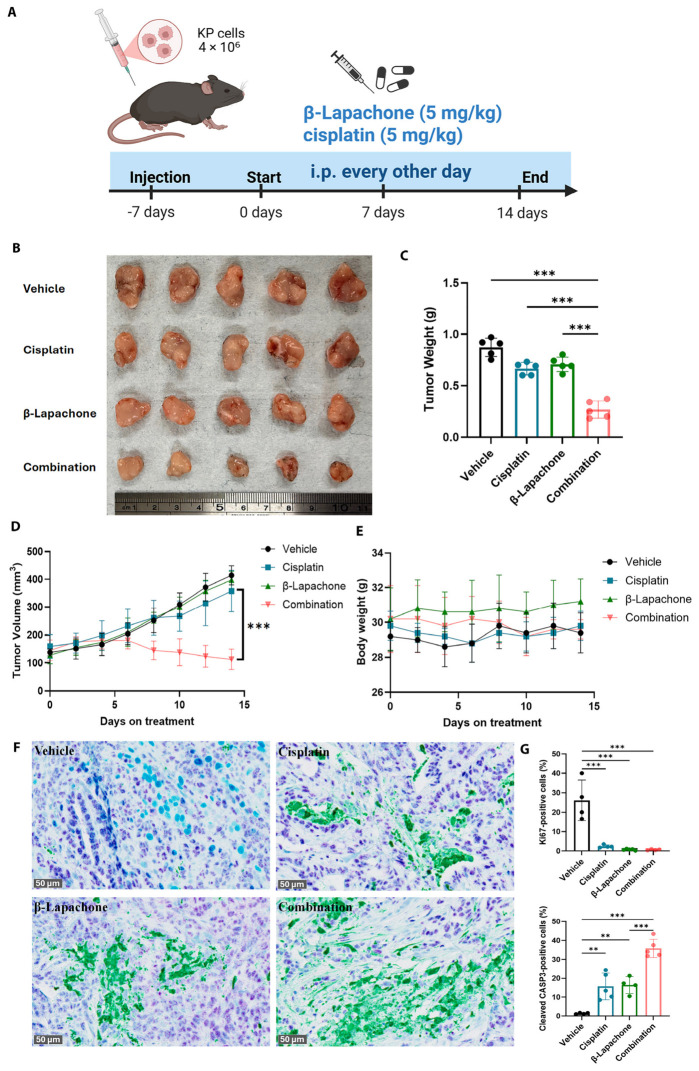
β-Lapachone potentiates the antitumor efficacy of cisplatin in vivo. (**A**) Schematic of the treatment regimen. Syngeneic KP tumor-bearing C57BL/6J mice were randomized to receive vehicle, cisplatin (5 mg/kg), β-Lapachone (5 mg/kg), or the combination by intraperitoneal injection every other day for 2 weeks. (**B**) Representative images of excised tumors at the experimental endpoint. (**C**) Tumor weights at sacrifice. Data represent the mean ± SD; *** *p* < 0.001, vehicle vs. treatment. (**D**) Tumor growth curves during treatment. (**E**) Body weight monitoring during treatment. (**F**) Representative immunohistochemical staining for USP22 (Purple), Ki67 (Turquoise) and cleaved caspase-3 (CC3, Green) in tumor sections (scale bar, 100 μM), ** *p* < 0.01, *** *p* < 0.001, vehicle vs. treatment. (**G**) Quantification of Ki67-positive and CC3-positive cells from ≥ 5 random fields per tumor.

As demonstrated in the above in vitro experiments, β-Lapachone may suppress lung cancer progression and enhance chemotherapy response, at least in part, through inhibition of USP22. To further validate these findings in vivo, we investigated whether the combination of β-Lapachone and cisplatin enhances antitumor efficacy using a syngeneic subcutaneous tumor model in C57BL/6J mice (Figure 6A). Compared with vehicle control and monotherapy, combination treatment produced the most pronounced suppression of tumor growth, as evidenced by significantly reduced tumor volume over the treatment period and lower tumor weights at the experimental endpoint (Figure 6C, D). Representative photographs of excised tumors further confirmed the superior antitumor response in the combination group (Figure 6B).

Body weight remained stable across all treatment groups throughout the study period, indicating that the combination regimen was well tolerated without overt systemic toxicity under the conditions tested (Figure 6E). To characterize treatment-associated changes in tumor biology, immunohistochemical analysis was performed on formalin-fixed tumor sections. Combination treatment significantly reduced the proportion of Ki67-positive proliferating cells and markedly increased the proportion of cleaved caspase-3 (CC3)-positive apoptotic cells relative to control and single-agent groups (Figure 6F,G). These histological findings are consistent with the in vitro data and confirm that β-Lapachone enhances the antitumor activity of cisplatin in vivo by suppressing tumor cell proliferation and promoting apoptosis.

### 2.7. Molecular Docking of β-Lapachone to Homology-Modeled Human USP22 DubM

**Figure 7:**
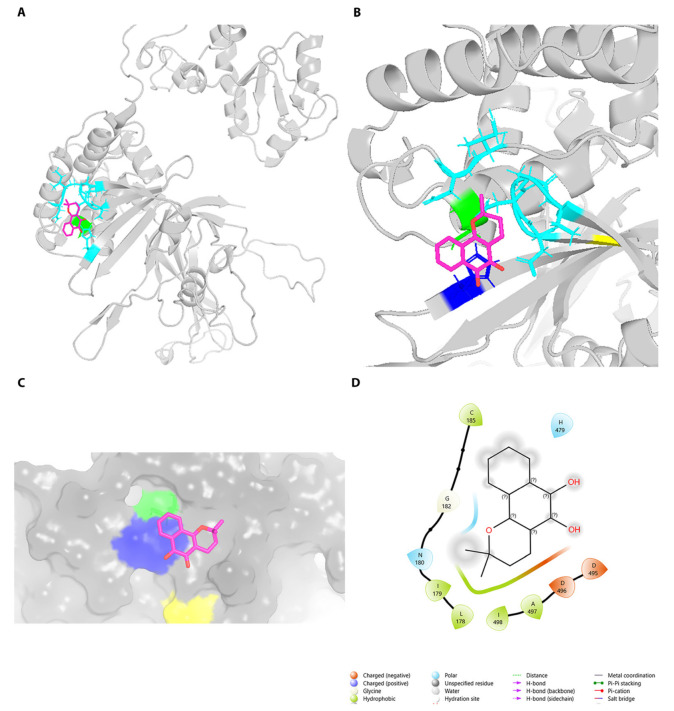
Molecular docking of β-Lapachone within the catalytic pocket of homology-modeled human USP22 DubM. (**A**) Global view of the human USP22 DubM model, generated using the yeast UBP8 crystal structure (PDB: 3MHS) as a template, shows overall fold of human USP22 DubM and β-Lapachone binding within the catalytic region. The protein is rendered as a gray cartoon with the ligand displayed as sticks (purple) and catalytic triad (cyan) residues color-coded to indicate the active site. Catalytic residues Cys185 (green), His479 (blue), and Asp493 (yellow) are highlighted throughout. (**B**) Binding pocket view highlighting residues within 5 Å of the ligand. (**C**) Surface cutaway representation of the binding cavity showing ligand burial and pocket topology. The semi-transparent surface highlights cavity shape, with the ligand positioned deep within the pocket and catalytic residues marking the active center. (**D**) Two-dimensional protein-ligand interaction diagram showing residues within 4.5 Å of the ligand. The binding environment is predominantly hydrophobic (Ile178, Ile179, Ala497, Ile498) with nearby acidic residues (Asp495, Asp496). No canonical hydrogen bonds are observed. The ligand is located near Cys185 without evidence of direct covalent or hydrogen-bond interaction.

To further elucidate the structural basis by which β-Lapachone acts as a potent inhibitor of USP22 DubM and suppresses its deubiquitinating activity, molecular modeling of the human USP22 DubM domain and protein-ligand interactions were conducted using bioinformatic approaches. Molecular docking was performed using a homology-modeled structure of human USP22 (Figure 7A) based on the 3MHS template to evaluate the binding mode of β-Lapachone within the catalytic site. β-Lapachone was consistently predicted to occupy the catalytic pocket adjacent to the catalytic triad residues Cys185, His479, and Asp493 [30,31,32]. As shown in Figure 7A, the model indicates that β-Lapachone localizes within the central catalytic region of USP22. The top docking pose showed a binding affinity of approximately −6.8 kcal/mol, indicating a stable and consistent binding orientation within the pocket.

Furthermore, visualization of the highest-scoring docking pose in PyMOL Version 4.60 (Figure 7B) showed that β-Lapachone occupies the predicted catalytic cavity of USP22 and is positioned adjacent to the catalytic triad residues Cys185, His479, and Asp493, confirming its localization within the active-site region of the homology model. Residues within 5 Å of the ligand define a well-formed binding pocket that encloses β-Lapachone, with the catalytic triad situated in proximity, further supporting its placement at the enzymatic core.

Surface cutaway analysis (Figure 7C) further indicates that β-Lapachone is partially buried within the binding cavity rather than exposed to solvent, consistent with stable pocket occupancy. At a 4.5 Å interaction cutoff (Figure 7D), the ligand is embedded in a compact hydrophobic environment composed primarily of Ile178, Ile179, Ala497, and Ile498, which shapes a nonpolar pocket accommodating the ligand scaffold. In contrast, Asp495 and Asp496 are located near ligand hydroxyl groups, suggesting a surrounding polar microenvironment; however, no stable hydrogen bonds are detected under standard geometric criteria.

Notably, Cys185 is near the ligand but does not form covalent or direct hydrogen-bond interactions, indicating that β-Lapachone binds adjacent to the catalytic thiol rather than engaging it directly. Overall, these results support a non-covalent binding mode driven mainly by hydrophobic interactions and steric complementarity, with electrostatic contributions from nearby polar residues. This binding configuration provides a structural basis for potential modulation of USP22 activity, warranting further validation by biochemical and biophysical studies.

## 3. Discussion

In this study, we established a potent and specific strategy to identify inhibitors of USP22 by directly interrogating the enzymatic activity of the intact SAGA DubM rather than relying solely on in silico predictions [30,31,32]. A major limitation in current USP22 drug discovery efforts is that USP22 lacks catalytic activity when isolated and requires assembly with its SAGA subunits for function [33]. Many previously reported inhibitors were identified using truncated or artificial systems, or through virtual docking approaches constrained by the lack of high-resolution structural data for the USP22-SAGA complex. By contrast, our fluorescence-based ubiquitin–rhodamine 110 assay reconstitutes the functional USP22 DubM, enabling direct biochemical measurement of enzymatic inhibition. Using this platform, we identified β-Lapachone as a USP22 inhibitor with an IC_50_ of ~0.75 μM, which is lower than or comparable to previously reported small-molecule USP22 inhibitors such as Rottlerin, Morusin [36] and Gentiopicroside [37]. The biochemical activity observed in a fully assembled USP22 complex suggests improved functional specificity and underscores the advantage of our assay system for discovering bona fide USP22 inhibitors with translational potential.

Although docking predicted only modest interactions between β-Lapachone and USP22 DubM, it potently suppresses deubiquitinase activity of USP22 DubM in vitro. The ligand was positioned near the catalytic Cys185, suggesting inhibition may occur through steric interference, transient or water-mediated interactions, or stabilization of an inactive enzyme conformation rather than strong canonical hydrogen bonds. This apparent discrepancy likely reflects the limitations of rigid docking models, which do not fully capture USP22 conformational dynamics or induced-fit effects [33]. Furthermore, the docking structure was based on a homology model of human USP22 derived from yeast UBP8 (~31.9% sequence identity) [30], which may not accurately represent the native active-site geometry [30,31,32]. Therefore, the strong biochemical inhibition observed experimentally supports a functionally relevant binding mode that is not fully reflected by docking predictions.

β-Lapachone is a well-characterized anticancer agent with pleiotropic biological activities, including topoisomerase I inhibition, NQO1-dependent redox cycling, induction of DNA damage, suppression of DNA repair [44], and modulation of oncogenic pathways such as mTOR and AKT signaling [45,46,47]. Previous studies demonstrated that β-Lapachone induces cell cycle arrest at the S and G2/M phases and apoptosis through both p53-dependent and p53-independent pathways [48,49]. Despite extensive investigation, the complete molecular target spectrum underlying its anti-tumor effects has remained incompletely defined. Our findings identify USP22 as a previously unrecognized and functionally relevant target of β-Lapachone, thereby integrating deubiquitinase inhibition into its known mechanism of action. In KRAS-mutant lung cancer models, β-Lapachone suppressed proliferation, induced apoptosis, and synergized with cisplatin to enhance DNA damage and tumor cell killing. Importantly, genetic ablation of USP22 conferred partial resistance to β-Lapachone, demonstrating that USP22 inhibition is a critical contributor to its anticancer activity. Mechanistically, β-Lapachone attenuated USP22-driven oncogenic signaling and impaired cancer stem cell properties, as evidenced by reduced sphere formation and decreased CD133^+^ cell populations. It is worth noting that β-Lapachone-mediated inhibition of USP22 may potentially influence other downstream targets of USP22, such as YAP, thereby contributing to its broader biological and antitumor effects. Given that USP22 has been reported to stabilize YAP by preventing its degradation [16], inhibition of USP22 by β-Lapachone in KRAS-mutant lung cancer may attenuate YAP/TEAD signaling, which plays a crucial role in KRAS-mutant lung cancer stemness, growth, survival, and therapeutic resistance [50,51]. Consequently, suppression of the USP22-YAP/TEAD axis could potentially contribute to the antitumor activity of β-Lapachone and may help limit resistance-associated mechanisms. However, whether β-Lapachone directly modulates YAP/TEAD signaling through USP22 inhibition in KRAS-mutant lung cancer remains to be experimentally determined. These findings position USP22 at the intersection of β-Lapachone–mediated cytotoxicity, therapy sensitization, and suppression of tumor stemness. The significance of identifying USP22 as a novel functional target of β-Lapachone extends beyond lung cancer biology. USP22 is a multifaceted oncogenic regulator implicated in tumor proliferation, growth, angiogenesis, epithelial–mesenchymal transition (EMT), and maintenance of cancer stemness across multiple cancer types [6,12,13,14,15]. More recently, USP22 has emerged as an important modulator of antitumor immunity, including roles in immune evasion and regulation of T regulatory cell infiltration within the tumor microenvironment [25,26,27]. Therefore, pharmacological inhibition of USP22 may simultaneously suppress intrinsic tumor aggressiveness and reshape immune responses to favor anticancer immunity. Our findings will thus further expand the application of β-Lapachone in cancer treatment as monotherapy particularly in combination with other regimens.

Our findings expand the therapeutic relevance of USP22 by providing a mechanistic explanation linking its anticancer and immunomodulatory effects. β-Lapachone exerts potent anticancer activity through NQO1-dependent ROS generation and PARP-mediated metabolic collapse [44]. This mechanism is particularly relevant in KRAS-mutant non-small-cell lung cancer, where NQO1 is frequently elevated in tumor tissues, rendering cancer cells selectively sensitive to NQO1-bioactivatable agents such as β-Lapachone [52,53,54]. In this context, we identify USP22 as a critical determinant of drug response, as USP22 knockout confers resistance to β-Lapachone, suggesting that USP22 status may influence therapeutic sensitivity and could serve as a predictive biomarker for patient selection.

Importantly, β-Lapachone treatment also suppresses cancer stemness and enhances sensitivity to conventional chemotherapy, including cisplatin, supporting the potential for combination strategies [18,28,29]. Although the clinical application of β-Lapachone has been limited by dose-dependent toxicities observed in early clinical studies [54], combination approaches may improve therapeutic efficacy while reducing the required drug exposure. In a Phase 1b clinical trial, β-Lapachone combined with gemcitabine and nab-paclitaxel improved outcomes in patients with advanced pancreatic cancer [55]. Another study showed that administering β-Lapachone after ionizing radiation hyperactivated PARP and increased DNA double-strand breaks, thereby enhancing cytotoxicity while reducing side effects in NSCLC models [56]. In this study, KRAS-mutant lung cancers with high USP22 expression are particularly sensitive to β-Lapachone, especially when combined with cisplatin, suggesting that USP22 may serve as a potential predictive biomarker for patient stratification. Given that USP22 is frequently overexpressed in multiple cancer types and is associated with tumor progression, stemness, and therapeutic resistance, it is conceivable that the therapeutic vulnerability identified here may extend beyond KRAS-mutant lung cancer. Thus, NQO1-high tumors with elevated USP22 expression may represent a subset of patients most likely to benefit from β-Lapachone-based therapy, either alone or in combination with cisplatin. Future studies are warranted to evaluate USP22 as a potential biomarker of β-Lapachone responsiveness across diverse malignancies.

## 4. Materials and Methods

### 4.1. Reconstitution of Human USP22 DubM and Fluorescence-Based USP22 Deubiquitinase Assay

The USP22 DUB complex (szHFF1), consisting of USP22 (residues 2–525; N-terminal 3×Flag-His-TEV tagged), ENY2 (residues 1–101), ATXN7L3 (residues 2–151; N-terminal Strep-TEV tagged), and ATXN7 (residues 75–172), which has been reported to constitute the minimal functional deubiquitinase module required for USP22 catalytic activity [33], was co-expressed in insect cells using two pFastBacDual constructs (szHFD1 and szHFE1) via baculoviral infection. Cells were harvested 48–72 h post-infection and lysed under native conditions. The complex was purified by Ni-NTA affinity chromatography via the His tag on USP22, followed by TEV protease cleavage to remove the tag. A secondary affinity step was performed to remove the protease and uncleaved material, and the complex was further purified by size-exclusion chromatography to yield a homogeneous, untagged complex. Deubiquitinase activity of the recombinant human USP22 complex was measured using a fluorogenic ubiquitin substrate. The USP22 complex was used at a final concentration of 0.25 nM. Assays were performed in reaction buffer containing 50 mm Tris-HCl (pH 7.5), 150 mm NaCl, 6 mm EDTA, 10 mm DTT, and 0.1% Tween-20 in black 384-well plates. The USP22 complex was first pre-incubated with 3-fold serial dilution of compounds (starting at 10 µM) or DMSO control (final DMSO ≤ 1%) for 20 min at 25 °C, and then reactions were initiated by addition of ubiquitin-Rhodamine 110 (Ub-Rho, from R&D Systems, Minneapolis, MN, USA) to a final concentration of 100 nM, incubated at 25 °C for 60 min. The relative fluorescence units (RFU) were monitored kinetically at 25 °C using a multimode microplate reader (Spark, Tecan, Männedorf, Switzerland) at excitation/emission wavelengths of 545/585 nm. Initial velocities were determined from the linear range of the fluorescence signal after subtraction of background (no enzyme control). Enzymatic activity was normalized to DMSO-treated controls, and IC_50_ values were calculated by nonlinear regression using a four-parameter logistic model (GradphPad Prism 11). The DUB assay was performed to screen the Library of Pharmacologically Active Compounds (LOPAC^®1280^, Sigma-Aldrich, St. Louis, MO, USA) for potent USP22 inhibitors.

### 4.2. Cell Proliferation and Compound Treatment

Human non-small-cell lung cancer cell lines A549 (p53 wild-type, KRAS-mutant) and H1299 (p53-null, HRAS-mutant) were purchased from ATCC and cultured in DMEM and RPMI-1640 medium, respectively, supplemented with 10% fetal bovine serum (FBS) and 100 U/mL penicillin and streptomycin. *USP22* knockout (*USP22*−/−, USP22-KO) cell lines were generated from A549 and H1299 cells using a CRISPR/Cas9 system as described previously [27]. All cell lines were authenticated and routinely tested to confirm the absence of mycoplasma contamination before use. For cell proliferation and cytotoxicity assays, cells were seeded in 96-well plates in 4–6 replicates at a density of 2.0 × 10^3^ cells per well. After 24 h, cells were treated with increasing concentrations of β-Lapachone (Cat#: HY-13555, MedChemExpress, Monmouth Junction, NJ, USA) or indicated compounds and incubated for an additional 72 h. Subsequently, the cells were washed once with phosphate-buffered saline (PBS), 100 μL of Calcein AM (2.5 μM, Cat#: C1430 Invitrogen, Carlbad, CA, USA) was added to each well, and the treated cells were incubated at 37 °C for 30 min protected from light. Fluorescence intensity was subsequently measured using a multimode microplate reader (Spark, Tecan, Männedorf, Switzerland) at excitation/emission wavelengths of 485/535 nm. Cell viability was normalized to that of vehicle-treated control cells and expressed as a percentage of control.

### 4.3. Stem Cell Sphere Formation and Colony Formation Assay

The human lung cancer stem-like cell lines LCSC-4 and LCSC-561912 were established in our laboratory from primary LUAD tissues obtained from patients [29]. Briefly, tumor tissues were dissociated into single-cell suspensions and cultured under serum-free, non-adherent conditions to enrich cancer stem-like cells with sphere-forming capacity. β-Lapachone and cisplatin (Cat#: HY-17394, MedChemExpress, Monmouth Junction, NJ, USA) were dissolved in DMSO and saline, respectively, at a 10 mm stock concentration. LCSC-4 and LCSC-561912 cells were seeded in a density of 500 cells/well of 6 wells plate and treated with increasing concentrations of β-Lapachone (0–4 μM) for 1 week. Sphere formation was assessed by microscopy using a 4× objective lens, and representative images were captured with a scale bar of 500 μM. Sphere size was quantified using ImageJ software, Version 1.54s, and data were expressed as sphere area normalized to the untreated control group. For clonogenic assays, A549 and H1299 cells were seeded in 6-well plates at low density (500 cells/well) and allowed to adhere overnight. Cells were then treated with β-Lapachone, cisplatin or their combination for 10–14 days, with medium refreshed every 3–4 days. At the endpoint, colonies were washed with PBS, fixed with 4% paraformaldehyde for 15 min, and stained with 0.1% crystal violet solution for 20 min at room temperature. Excess stains were removed by washing with distilled water, and plates were air-dried before imaging. Colony area was quantified using ImageJ software and expressed as a percentage relative to the untreated control.

### 4.4. Western Blot Analysis and Apoptosis Analysis

Cells were treated under indicated conditions and lysed in lysis buffer (R&D Systems, Minneapolis, MN, USA) supplemented with protease inhibitors. Protein concentrations were determined by BCA assay, and equal amounts of protein were separated by SDS-PAGE and transferred to PVDF membranes. Membranes were incubated with primary antibodies against USP22, ALDH1A3, c-Myc, p21, γ-H2A.X (phosphor S139), total and cleaved PARP, GAPDH, p53, acetylated p53 (Lys382), etc., purchased from Abcam (Cambridge, MA, USA), Cell Signaling Technology (Danvers, MA, USA), or Santa Cruz Biotechnology (Santa Cruz, CA, USA), followed by appropriate HRP-conjugated secondary antibodies. Protein bands were visualized using enhanced chemiluminescence (ECL). Band intensities were quantified using ImageJ software (NIH). After background subtraction, the intensity of each target protein band was normalized to GAPDH. Relative protein expression was calculated by comparing normalized values to those of the control group, which was set to 1. For phosphorylated proteins, band intensities were first normalized to their corresponding total protein levels and subsequently expressed relative to the control group.

Apoptosis was evaluated by flow cytometry using the Invitrogen™ Tali™ Apoptosis Kit (Thermo Fisher Scientific, Waltham, MA, USA) according to the manufacturer’s instructions. Briefly, after 48 h of treatment with β-Lapachone, cisplatin, or their combination, cells were harvested, washed twice with ice-cold PBS, and resuspended in 1× Annexin V binding buffer. For staining, approximately 1 × 10^5^ cells were incubated with Annexin V–Alexa Fluor 488 (final concentration ~5 µL per 100 µL cell suspension) and propidium iodide (final concentration ~1 µg/mL) for 15 min at room temperature in the dark. After incubation, an additional 400 µL of binding buffer was added, and samples were immediately analyzed by flow cytometry. Cells were classified as viable (Annexin V^−^/PI^−^), early apoptotic (Annexin V^+^/PI^−^), late apoptotic (Annexin V^+^/PI^+^), or necrotic (Annexin V^−^/PI^+^) based on fluorescence staining patterns.

### 4.5. Immunocytochemistry/Immunofluorescence Staining of γH2A.X (Phospho S139)

Cells were fixed with 4% paraformaldehyde and permeabilized with 0.1% Triton X-100 prior to blocking and antibody incubation. Immunofluorescence staining was performed using an anti-γH2A.X (phospho S139) antibody (1:250) to detect DNA damage foci. After washing, cells were incubated with an Alexa Fluor^®^ 488-conjugated goat anti-rabbit IgG secondary antibody (1:1000). Nuclei were counterstained with 0.2 μg/mL DAPI. Fluorescence images were acquired using a fluorescence microscope.

### 4.6. In Vivo Tumor Models and Treatment

This study was reviewed and approved by the Institutional Animal Care and Use Committee (IACUC, protocol #16,005) of City of Hope National Medical Center. All animal procedures were conducted in the animal facility at City of Hope National Medical Center in strict accordance with federal, local, and institutional guidelines. Murine primary KRAS^G12C^, p53-null lung cancers were generated using conditional KP mice (KRAS^LSL-G12C^; p53^flox/flox^) on a C57BL/6J background. After tumor formation, lung tumors were harvested under sterile conditions, mechanically dissociated, and cultured to establish murine KP lung cancer cell lines.

For syngeneic tumor studies, KP cells were harvested during the logarithmic growth phase, washed twice with sterile PBS, and resuspended in a 3:1 mixture of RPMI-1640 and Matrigel. A total of 4 × 10^6^ cells in 100 μL were injected subcutaneously into the flanks of 6–8-week-old C57BL/6J mice (equal numbers of males and females). Tumor growth was monitored twice weekly using digital calipers, and tumor volume was calculated as: V = (length × width^2^)/2. When the mean tumor volume reached approximately 100 mm^3^, mice were randomized into four treatment groups (n = 5 per group): vehicle control, cisplatin, β-Lapachone, and combination. No statistical methods were used to predetermine sample size. β-Lapachone (5 mg/kg) was dissolved in a vehicle containing 10% DMSO, 40% PEG300, 5% Tween-80, and 45% saline, cisplatin (5 mg/kg) were dissolved in sterile saline and administered by intraperitoneal (i.p.) injection every other day for 2 weeks, either as single agents or in combination. Control mice received equivalent volumes of vehicle. Body weight and tumor dimensions were recorded twice weekly. All animals were euthanized before tumors reached 3000 mm^3^ or showed signs of impending ulceration. Mice were euthanized after 2 weeks of treatment; tumors were excised, weighed, and photographed. Sections were subjected to IHC stains of Ki67 (cell proliferation marker) and cleaved caspase-3 (apoptosis marker), as described previously [27]. Stained sections were scanned and imaged under a light microscope. Quantification of Ki67- and CC3-positive cells was performed in at least five randomly selected high-power fields per tumor section using ImageJ software, and the percentage of positive cells was calculated relative to the total number of tumor cells.

### 4.7. Homology Modeling and Molecular Docking

Because no experimentally determined structure of human USP22 is currently available, a structural model of the USP22 catalytic domain in complex with ubiquitin was generated by homology modeling using the yeast UBP8 crystal structure (PDB ID: 3MHS) as a template [30]. This template was selected due to its structural and functional conservation within the SAGA deubiquitinase module. Sequence alignment revealed 31.86% identity between human USP22 and UBP8. The homology model was generated using the SWISS-MODEL server [57], and the final model was selected based on GMQE and QMEAN quality scores for downstream analyses.

The modeled structure was prepared for docking by removing non-protein atoms and crystallographic artifacts, followed by addition of polar hydrogens and assignment of atom types. The ligand β-Lapachone was energy-minimized and prepared with defined rotatable bonds to allow conformational sampling during docking. Molecular docking was performed using AutoDock Vina, Version 1.2.7#0 [58] to predict the binding mode of β-Lapachone within the catalytic pocket of USP22. The docking grid was centered on the catalytic triad residues Cys185, His479, and Asp493 and defined to encompass the full substrate-binding cleft. Multiple binding poses were generated and ranked according to predicted binding affinity. Top-ranked docking poses were analyzed to evaluate ligand orientation and interaction patterns within the catalytic site. Structural visualization was performed using PyMOL, Version 4.60 [59], and protein-ligand interaction profiling was conducted using Maestro (Release 2026-1, Schrödinger Suite). Distance-based criteria (≤5 Å) were applied to identify contact residues and hydrogen-bonding interactions. Particular attention was given to interactions near the catalytic cysteine (Cys185), given its central role in deubiquitinase activity. Collectively, the docking and interaction analyses suggest that β-Lapachone occupies the catalytic cleft of USP22 and forms stabilizing contacts within the active-site environment, consistent with potential inhibition of enzymatic activity.

### 4.8. Statistical Analysis

Statistical analyses were performed using GraphPad Prism 10.5.0. Data are presented as the mean ± standard deviation (SD). Comparisons between two independent groups were analyzed using unpaired Student’s *t*-tests. For experiments involving more than two groups, one-way or two-way analysis of variance (ANOVA) was applied as appropriate.

## 5. Conclusions

In conclusion, our study identifies β-Lapachone as a potent inhibitor of USP22 deubiquitinase activity and demonstrates that USP22 inhibition represents an important mechanism underlying its anticancer effects in RAS-mutant LUAD. RAS-mutant LUAD with high USP22 and NOQ1 expression may be especially responsive to β-Lapachone therapy, alone or with cisplatin.

## Data Availability

The original contributions presented in this study are included in the article. Further inquiries can be directed to the corresponding authors.
